# Commencement Exercises of the Medical Department of the University of Buffalo

**Published:** 1856-03

**Authors:** 


*Commencement Exercises of the Medical Department of the University of Buffalo.*—These were held at Kremlin Hall, on the evening of Feb. 26. The opening prayer, by the Rev. Mr. Heacock, was an impressive and appropriate appeal to the Almighty Power.

The Hon. Geo. W. Clinton, President of the Board of Trustees, gave the opening address, in the absence of the Chancellor, His Excellency Millard Fillmore.

Mr. Clinton spoke with fervor of the school and its usefulness. He hoped that the day was not distant when the other departments of the University should be added; testified to the entire confidence reposed in the Faculty by the Council, and stated, lest misapprehension should arise, that though, from various causes, the graduating class was unusually small, yet the number of matriculants was larger than that of the preceding year. He also made eloquent mention of Drs. J. Marion Sims, of New York city, and Jas. D. Trask, of White Plains, N. Y., who received honorary degrees as marks of the eminent appreciation in which they were held by the Council and Trustees, for services rendered to humanity. An honorary degree thus granted, without the knowledge or solicitation of the recipients, was a testimonial to their merit, which, though not needed by these distinguished gentlemen, would not be unvalued.

Mr. Clinton closed by an eloquent tribute to the gallant physicians who faced the pestilence at Norfolk, and to Florence Nightingale, the heroine of the Crimea.

He then conferred the degree on the several graduates.

Prof. James P. White delivered the charge to the graduates. It was a gracefully written and effective effort, and absorbed the attention of the auditory during its delivery. Without attempting to wander far from the beaten path of all similar addresses, his remarks were replete with sound counsel to the class, as to the choice of location, their duties to society, to the profession, and to themselves. He enforced the importance of an educated mind, in the true sense of the term as distinguished from the mere acquirement of facts, and forcibly pointed out that knowledge alone is not power, but requires a competent and trained intellect to govern and guide its application to practical matters. While he did not undervalue the practical, executive mind, he administered an indignant rebuke to those drones in the profession, who are constantly croaking against its more learned members as mere "book-worms," good students, good writers, or good teachers, but not good practitioners. He demonstrated the folly of this reasoning, and carried the war into Africa by an exposure of the habits of mind and action of the routinist.

Prof. White closed with an injunction to the class to strive to do honor to themselves and thus to honor their Alma Mater.

The Benediction was then pronounced by Rev. Mr. Heacock.

The exercises were interspersed with excellent music.

